# DHT inhibits REDOX damage and neuroinflammation to reduce PND occurrence in aged mice via mmu_circ_0001442/miR‐125a‐3p/NUFIP2 axis

**DOI:** 10.1002/brb3.3180

**Published:** 2023-08-07

**Authors:** Li Liu, Mei Liu, Daying Zhang, Zhiping Song, Huaigen Zhang

**Affiliations:** ^1^ Department of Oncology Jiangxi Provincial People's Hospital Nanchang Jiangxi P. R. China; ^2^ Department of Anesthesiology The First Affiliated Hospital of Nanchang University Nanchang Jiangxi P. R. China; ^3^ Department of Pain Management The First Affiliated Hospital of Nanchang University Nanchang Jiangxi P. R. China

**Keywords:** aged mice, dihydrotestosterone (DHT), mmu_circ_0001442, neuroinflammation, perioperative neurocognitive disorder (PND), REDOX damage

## Abstract

**Background:**

Perioperative neurocognitive disorder (PND) is the main cause of poor postoperative recovery in elderly patients with age‐related reductions in androgen levels. However, the underlying mechanisms have not been completely elucidated.

**Methods:**

A mouse model of PND was constructed using abdominal surgery. Dihydrotestosterone (DHT), as the primary androgen, can improve the cognitive function of mice with PNDs by reducing REDOX damage. To clarify the role of circular RNA (circRNA) in DHT in improving cognitive function in mice with PND, circRNA sequencing was performed to analyze the expression of circRNA in the hippocampus of mice.

**Results:**

We confirmed that mmu_circ_0001442 is the primary circRNA responsive to DHT stimulation in mice with PND. The mmu_circ_0001442/miR‐125a‐3p/NUFIP2 axis was predicted and constructed according to the analysis of databases, including pita, miRanda, TargetScan, miRDB, micro‐CDS, PolymiRTS, and TarBase v.8. Subsequently, the axis was verified by qPCR and double‐luciferase reporter gene assays. In vitro, we found that DHT rarely had an effect on the growth of BV2 cells using the CCK‐8 assay, but it attenuated the cytotoxic effect of lipopolysaccharide (LPS) on BV2 cells. In addition, we found that LPS stimulation promoted the release of proinflammatory cytokines, including IL‐6 and TNF‐α, in BV2 cells, whereas mmu_circ_0001442 knockdown and NUFIP2 knockdown partially abrogated this effect.

**Conclusions:**

Taken together, DHT inhibited REDOX damage and neuroinflammation in the hippocampus to alleviate cognitive disorders in mice with PNDs via activation of the mmu_circ_0001442/miR‐125a‐3p/NUFIP2 axis. This study provides a novel rationale for developing DHT as a potential therapeutic agent for PND prevention.

## Graphical abstract

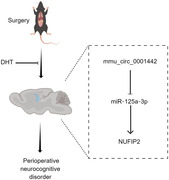

## 1 INTRODUCTION

Perioperative neurocognitive disorder (PND) is a common central nervous system (CNS) injury in elderly patients after surgery (Evered et al., 2018; Luo et al., 2019). A study reported that the maximum incidence of delirium (as a usual symptom of PND) postoperatively occurred in 65% of elderly patients (Vacas et al., 2021). PND usually manifests as mental decline, memory loss, inattention, abstract thinking, orientation, and sleep disorders (Jin et al., 2020). All in all, PND may bring about a decrease in social activities and even increase postoperative mortality in elderly patients. Therefore, elucidating the pathogenesis of PND is beneficial to solve the problem of poor recovery in elderly patients. Age‐related reduction in androgen levels is an important risk factor leading to the occurrence of cognitive disorder (Cai & Li, 2020; McHugh et al., 2018). However, whether low level of androgens is the key cause of the high incidence for PND in elderly patients still needs further research.

As the agonists of the androgen receptor (AR), androgens are natural or synthetic steroids mainly including testosterone and dihydrotestosterone (DHT) (Schiffer et al., 2018). Current studies have shown that 10 nM DHT in vitro and 5 mg/(kg/day) DHT in mice have anti‐neuroinflammatory and neuroprotective effects (Yang et al., 2020). In addition, DHT deficiency can increase REDOX damage and reduce synaptic plasticity, which resulting in cognitive disorder (Spence & Voskuhl, 2012). Circular RNA (circRNA), as a valuable noncoding RNA, can exert sponge effect in regulating target gene. For example, a study identified a ceRNA network and target gene of aged mice suffering PND using NGS technology and further predicted some small‐molecule drugs with potential therapeutic effects on PND (Wu et al., 2021). High‐throughput sequencing analysis of brain tissue from PND model mice is considered to be a favorable strategy to study the mechanism of PND. Two important ceRNA regulatory networks were identified including mmu_circ_0000331/miR‐1224‐3p/Unc13c and mmu_circ_0000406/miR‐24‐3p/St8sia2 ceRNA networks via high‐throughput sequencing analysis (Bao et al., 2022). However, there is still a lack of therapeutic measures targeting the PND‐related ceRNA regulatory network. Studying the anti‐neuroinflammatory effect of DHT by regulating noncoding RNA can provide a novel perspective to understand the mechanism of neuroinflammation and to better develop therapeutic agents of PND.

There is no doubt that neuroinflammation exerts a robust effect in the occurrence of PND (Saxena et al., 2021; Subramaniyan & Terrando, 2019). Current studies suggest that both anesthesia and surgery can induce neuroinflammation in the CNS, which resulting in worse cognitive function (Evered et al., 2018). Notably, as a unique resident immune cell in the CNS, aberrant activation of microglia is a major feature of neuroinflammation. Activated microglia can promote the release of multiple neurotoxic mediators and proinflammatory cytokines, resulting in progressive neuronal cells death and the occurrence of PND (Cai & Li, 2020; Leitner et al., 2019; Zhao et al., 2021). The above neurotoxic mediators mainly include cyclooxygenase‐2 (COX‐2) and inducible nitric oxide synthase (iNOS) (Amor et al., 2014; Kwon & Koh, 2020). In addition, the above proinflammatory cytokines, including IL‐6 and TNF‐α, are positively connected with the occurrence of PND (Hu et al., 2018; Zhao et al., 2021). Lipopolysaccharide (LPS), an endotoxin composed of O‐antigens, is reported to be the most potent stimulator of microglia activation (Henry et al., 2009). However, there are few research focusing on the effect of DHT in reducing PND occurrence in aged mice by inhibiting neuroinflammation especially in microglia.

In order to elucidate the pathogenesis of PND, first, aged mice were selected as experimental animals, and abdominal surgery was performed to establish PND model in aged mice. Behavioral assays were used to determine the occurrence of PND in aged mice. Finally, this article found an identified ceRNA network playing a key role in anti‐neuroinflammatory and neuroprotective effects of DHT stimulation in PND mice. In a word, this article elucidated the mechanisms of PND and developed DHT as a potential medicine for PND prevention.

## 2 METHODS AND MATERIALS

### 2.1 Cell line culture

Mice brain microglial cell BV2 (KG645) was purchased from KeyGEN BioTECH, cultured in 1640 medium supplemented with 1% penicillin and streptomycin mixture, 10% fetal bovine serum. In addition, NIH‐3T3 cells were purchased from ATCC, cultured in DMEM medium supplemented with 1% penicillin and streptomycin mixture, 10% calf bovine serum with iron‐fortified. All above cells were maintained at 37°C in a humidified incubator.

### 2.2 Animal experiments

The all experiments were carried out in line with the experimental animal specifications of ZHBY Biotech Co. Ltd and were permitted by the Experimental Animal Welfare and Ethics Committee of ZHBY Biotech Co. Ltd (approval number: 2020052001). Animal experiments were performed in line with the revised Animals (Scientific Procedures) Act 1986 in the United Kingdom and Directive 2010/63/EU in Europe. Being purchased from an acceptable company, the 14‐month‐old healthy male C57BL/6 mice were fed and experimented in the SPF room. These mice were randomly divided into two groups using a random numbers table: DHT pretreatment group (T group, including 10 mice) and control group (C group, including 10 mice). In addition, the estimation of mice number was based on the law of diminishing returns. Mice in the T group were subcutaneously injected with DHT dissolved in corn oil on the nape of the neck at the dose of 1 mg/(kg/day). In addition, mice in the C group were injected with the same dose of medical corn oil. The entire injection processes lasted 14 days and were given once a day in the morning. Subsequently, PND model of mice was established by means of abdominal surgery. Notably, the study followed ARRIVE guidelines 2.0 (https://arriveguidelines.org/resources/author‐checklists).

### 2.3 Preparation of PND model

First of all, mice were anesthetized with isoflurane, and the disappearance of leg retraction and eyelid reflexes indicated that the depth of anesthesia was required for surgery. And then, after sterilizing the operation area using chlorhexidine, a 2 cm long incision was operated in the middle of the abdomen of the mice. Subsequently, the small intestine about 10 cm long was pulled out, and the abdominal cavity was explored. Notably, mice were randomly selected for operations. After completing these operations, the small intestine was placed back to the abdominal cavity and then sutured the wound. In addition, 0.2% ropivacaine was locally infiltrated on the wound for postoperative analgesia. It is necessary to complete the entire operation within 60 min. During the operation, the intestine temperature of the mice was monitored and was maintained at 36.9 ± 0.4°C using a heating lamp. The vital signs, such as mean arterial pressure, arterial oxygen saturation, and pulse, were continuously monitored. Notably, novel object recognition (NOR) detection and the expression of proteins of REDOX damage were used to evaluate the PND model.

### 2.4 Novel object recognition (NOR)

In order to define the effect of DHT on protecting cognitive functions, NOR was used to evaluate the nonspatial working memory abilities of animals (Saxena et al., 2021). The NOR consists of three stages: adaptation period, familiarity period, and testing period. First of all, each mouse was placed in an empty behavior box (50 × 50 × 60 cm^3^) for 5 min/day during 2 days to acclimate to the environment. Subsequently, three objects with a constant weight were prepared including two identical objects and another object of different shape. After entering the familiarity period, both of two identical objects and mice were placed in the behavior box. A timer was used to record the time of the mice exploring each object. On the third day after the abdominal surgery, the time spent exploring another novel object was recorded again. Notably, exploratory behavior was defined as the nose coming to within 1 cm of the object. The exploration times of mice to new and old objects were, respectively, recorded as TN and TF, and finally, the recognition index (RI) was calculated as RI = TN/(TF + TN).

### 2.5 Cell viability assay

The effect of DHT on the proliferation of BV2 cells was determined using the CCK‐8 assay. BV2 cells in the logarithmic phase, first, were seeded in a 96‐well plate at a density of 2×10 (Vacas et al., 2021) cells/well. After 24 h, DHT was intervened with a continuous concentration gradient (0, 2, 4, 6, 8, and 10 nM) for 12, 24, and 48 h, and then 10 μL CCK‐8 solution was added to a 96‐well cell culture plate and the incubation was continued for 0.5–4 h in an incubator at 37°C. Absorbance was measured at a wavelength of 450 nm.

### 2.6 Colony formation assay

To investigate the effect of DHT on BV2 cells growth, colony formation assay was carried out. BV2 cells in the logarithmic phase were seeded in a 6‐well plate at a density of 1 × 10 (Vacas et al., 2021) cells/well. After treatments, BV2 cells were fixed with 4% paraformaldehyde. Then, colonies were stained with coomassie blue solution.

### 2.7 qPCR

Total cellular RNA was extracted with RNAiso Plus. After removal of genomic DNA with the gDNA kit, targeted genes were analyzed using Invitrogen SuperScript IV One‐Step RT‐PCR System. Relative expression levels of cellular genes were calculated using the 2^−ΔΔ^ *^CT^* method.

### 2.8 Western blotting

BV2 cells in logarithmic growth phase were washed with PBS, then 100 μL RIPA cell lysate was added to each well. The lysis mixture solution transferred to a 1.5 mL EP tube. After centrifugation at 12,000 rpm at 4°C for 20 min, the supernatant was collected as total cell protein. The proteins were separated by SDS–polyacrylamide gel electrophoresis and identified by antibody antigen binding. Finally, the proteins were visualized by ECL chemiluminescence. The primary antibodies included COX‐2 (4842S, CST), iNOS (13120S, CST), and GAPDH (5174S, CST).

### 2.9 ELISA assay

The concentrations of proinflammatory cytokines IL‐6 (KMC0061, Invitrogen) and TNF‐α (BMS607‐2INST, Invitrogen) in the hippocampus of aged mice were detected using an ELISA assay. The samples were stored in an −80°C refrigerator. The supernatant of the ground samples was used to detect the concentration of IL‐6 and TNF‐α.

### 2.10 Hematoxylin–eosin staining (HE)

Hematoxylin–eosin (HE) staining was executed with a hematoxylin–eosin staining kit (G1120, Solarbio). Briefly, on the third day after the operation, the hippocampus tissue was removed. After dewaxing, 10% hematoxylin and 1% eosin were used to stain the Nuclei and cytoplasm, respectively. Digital images of stained sections were acquired using a LSM710 confocal microscope (Zeiss, Jena, Germany).

### 2.11 CircRNA sequencing

To clarify the role of circRNA in DHT improving cognitive function in mice with PND, circRNA sequencing was preformed to analyze the expression of circRNA in hippocampus of mice. The hippocampus tissue of representative mice was extracted in the testosterone group and the control group. Gene abundance was calculated and normalized by the SRPBM (spliced reads per billion mapping) method. The DESeq2 (V1.6.3) of the Bioconductor software package was used to identify differentially expressed circRNA between DHT‐treated and control groups. Significantly different genes were those with (log FC) ≥2 and *p*‐value <.05.

### 2.12 Double luciferase reporter gene assay

The dual‐luciferase reporter gene vector was customized and synthesized by Shenggon company (Sangon Biotech Co. Ltd). Likewise, the dual‐luciferase reporter vector containing the wild type (WT) or mutant (Mutant type [MT]) of mmu_circ_0001442‐3′ UTR sequence and the mimics of miR‐125a‐3p were both constructed by Shenggon company (Sangon Biotech).

### 2.13 Bioinformatics analysis

The regulatory network of mmu_circ_0001442 was analyzed using a series of databases. In particular, these databases include pita (http://genie.weizmann.ac.il/pubs/mir07/mir07_dyn_data.html), miRanda (http://www.bioinformatics.com.cn/), TargetScan (http://www.targetscan.org/), miRDB (http://www.mirdb.org/), micro‐CDS (https://dianalab.e‐ce.uth.gr/html/dianauniverse/index.php?r=microT_CDS), PolymiRTS (https://compbio.uthsc.edu/miRSNP/), and TarBase v.8 (https://dianalab.e‐ce.uth.gr/html/diana/web/index.php?r=tarbasev8).

### 2.14 Statistical analysis

Statistical analysis was performed using SPSS 22.0 software. Independent sample *t* test or one‐way analysis of variance was used to analyze independent measurement data of two groups that satisfy normality and homogeneity of variance at the same time. The Bonferroni method was used for pairwise comparisons. Differences were considered statistically significant when *p* ≤ .05.

## 3 RESULTS

### 3.1 DHT inhibited REDOX damage and neuroinflammation in aged mice with PND

**FIGURE 1:**
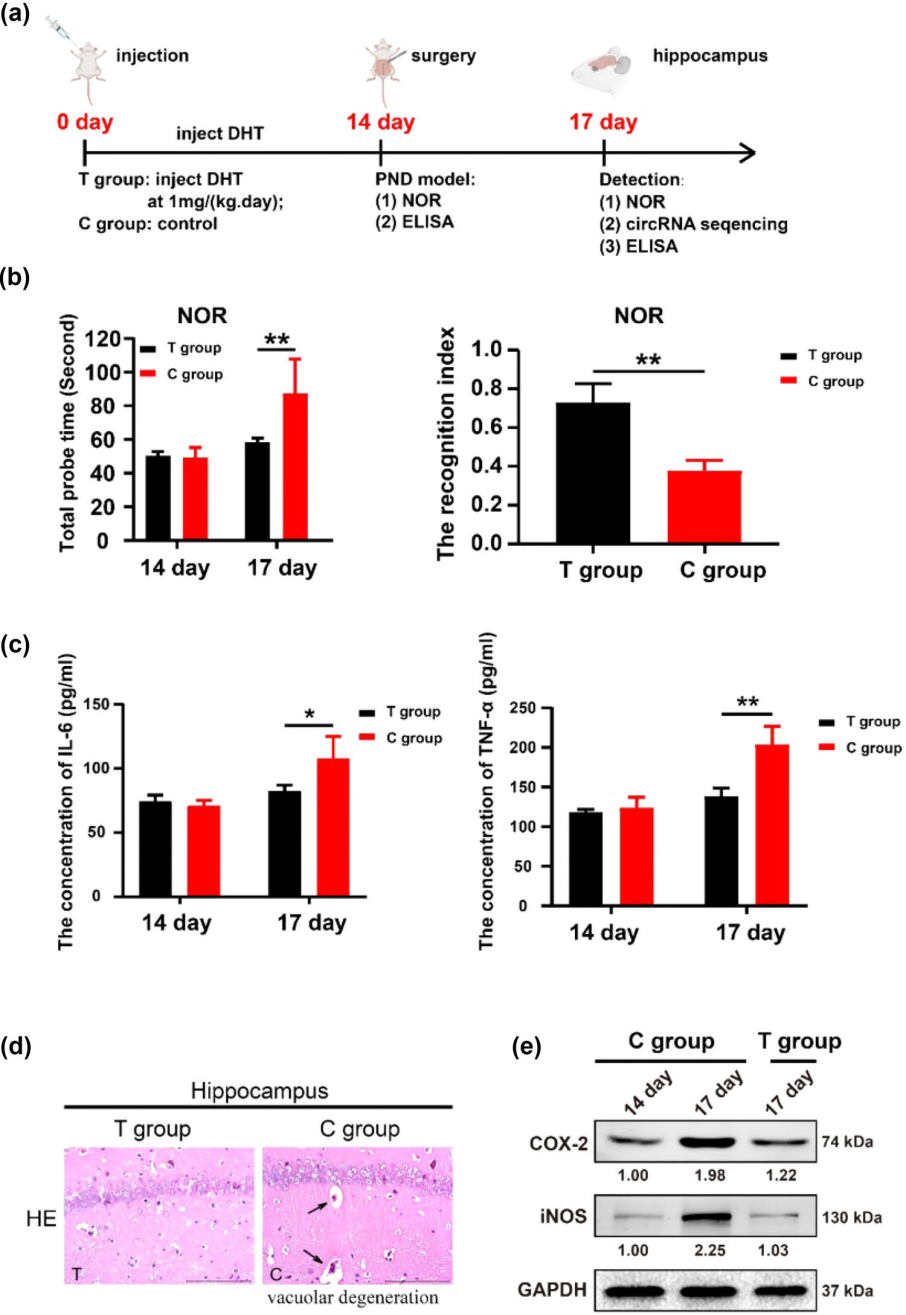
Dihydrotestosterone (DHT) inhibited REDOX damage and neuroinflammation in aged mice with perioperative neurocognitive disorder (PND). (a) The framework of animal experiment design (*n* = 10). (b) the total exploration time and the novel object recognition (NOR) were used to assess the cognitive function of aged mice. (c) ELISA kit, the concentrations of proinflammatory cytokines including IL‐6 and TNF‐α in the single‐cell suspension of hippocampus culture supernatant were detected using ELISA kit. (d) hematoxylin–eosin (HE) assays suggested milder inflammation of hippocampus than that in T group; Arrow indicated vacuolar degeneration. E, Western blotting detected the proteins of REDOX damage. All values are described as the mean ± SD. *p < .05, **p < .01. circRNA, circular RNA.

Accumulating evidence suggested that REDOX damage is strongly associated with a series of neurodegenerative and nervous system diseases (Steinert & Amal, 2023). In order to understand the improvement effect of DHT on spatial learning and memory in PND mice, the overall framework of animal experiments is shown in Figure 1a. The mice were divided into two groups including T group (DHT pretreatment group) and C group (control group). NOR was used to assess the cognitive function of aged mice at three moments including before abdominal surgery, and the third day after surgery. As shown in Figure 1b, the total exploration times of mice in the C group were higher than that in the T group on the third day after the operation; likewise, the RI of mice in the C group was lower than that in the T group on the third day after the operation. Current research showed that changes in cognitive function can be reflected in the neuroinflammation of the hippocampus (Saxena et al., 2021; Subramaniyan & Terrando, 2019). Two proinflammatory cytokines, including IL‐6 and TNF‐α, were identified as indicators of neuroinflammation. So, the expression trends of IL‐6 and TNF‐α in hippocampus were basically consistent with neuroinflammation features. In the T group, as DHT pretreatment group, the expression levels of IL‐6 and TNF‐α were significantly lower than that in C group (Figure 1c). As shown in Figure 1d, HE staining showed that the loosely dispersed neurons in the hippocampal CA1 region of the control group were accompanied by diffuse vacuolar degeneration and cell swelling, whereas the T group had remarkable improvement. Notably, Western blotting results suggested that DHT treatment can reduce the REDOX damage caused by the stimulation of abdominal surgery, manifested as the decreased expression of COX‐2 and iNOS (Figure 1e). Hence, the above results indicated DHT can inhibit REDOX damage as well as the expression of IL‐6 and TNF‐α in hippocampus of PND mice.

### 3.2 mmu_circ_0001442 was the primary circRNA responsive for DHT stimulation

**FIGURE 2:**
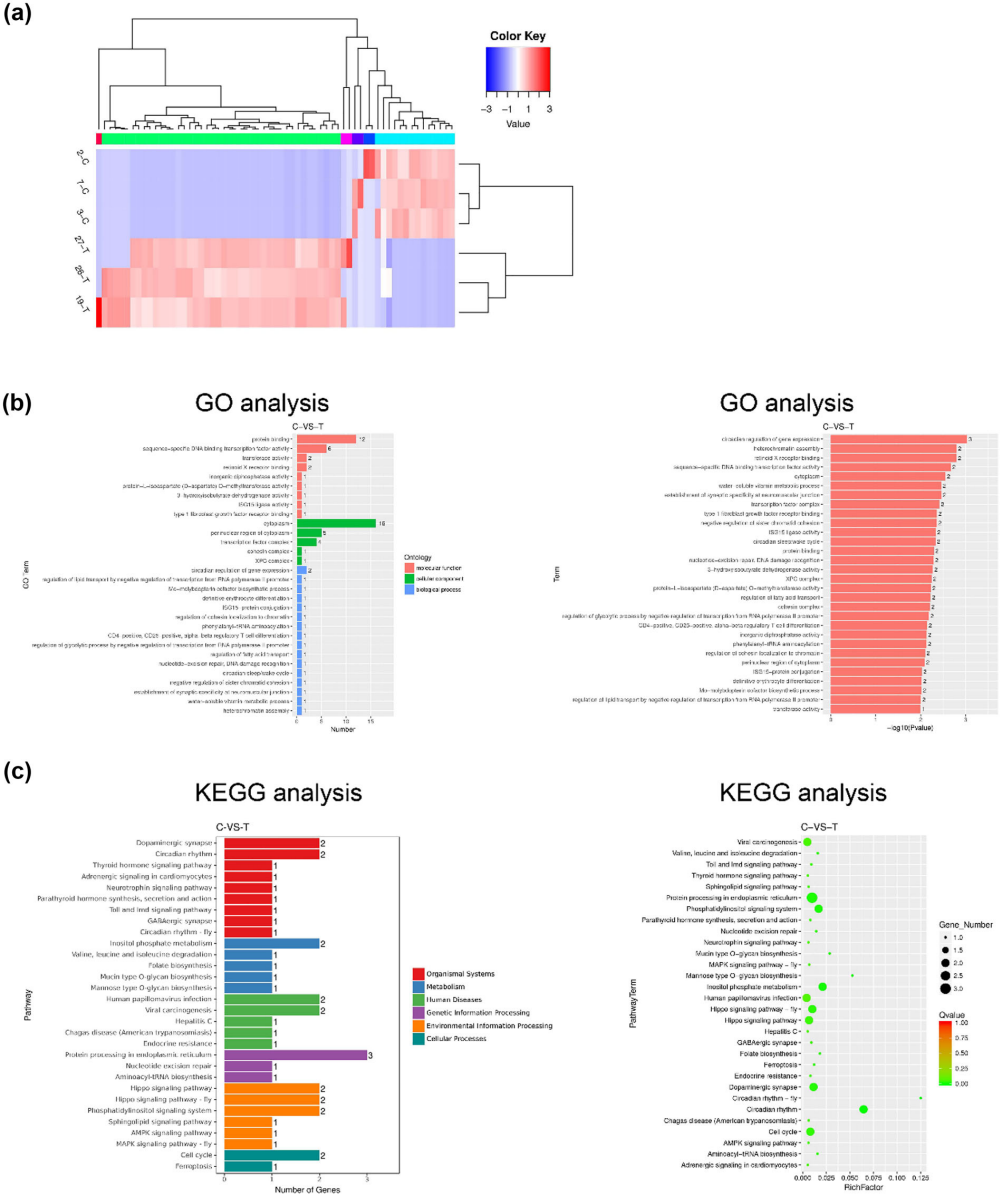
Circular RNA (circRNA) sequencing was preformed to analyze the effect of dihydrotestosterone (DHT) stimulation in hippocampus. (a) Heatmap was preformed to show the differentially expression circRNA between the T group and the C group. (b and c) GO and KEGG analysis was used to probe the function of the differentially expression circRNA. All values are described as the mean ± SD. *p < .05, **p < .01.

**FIGURE 3:**
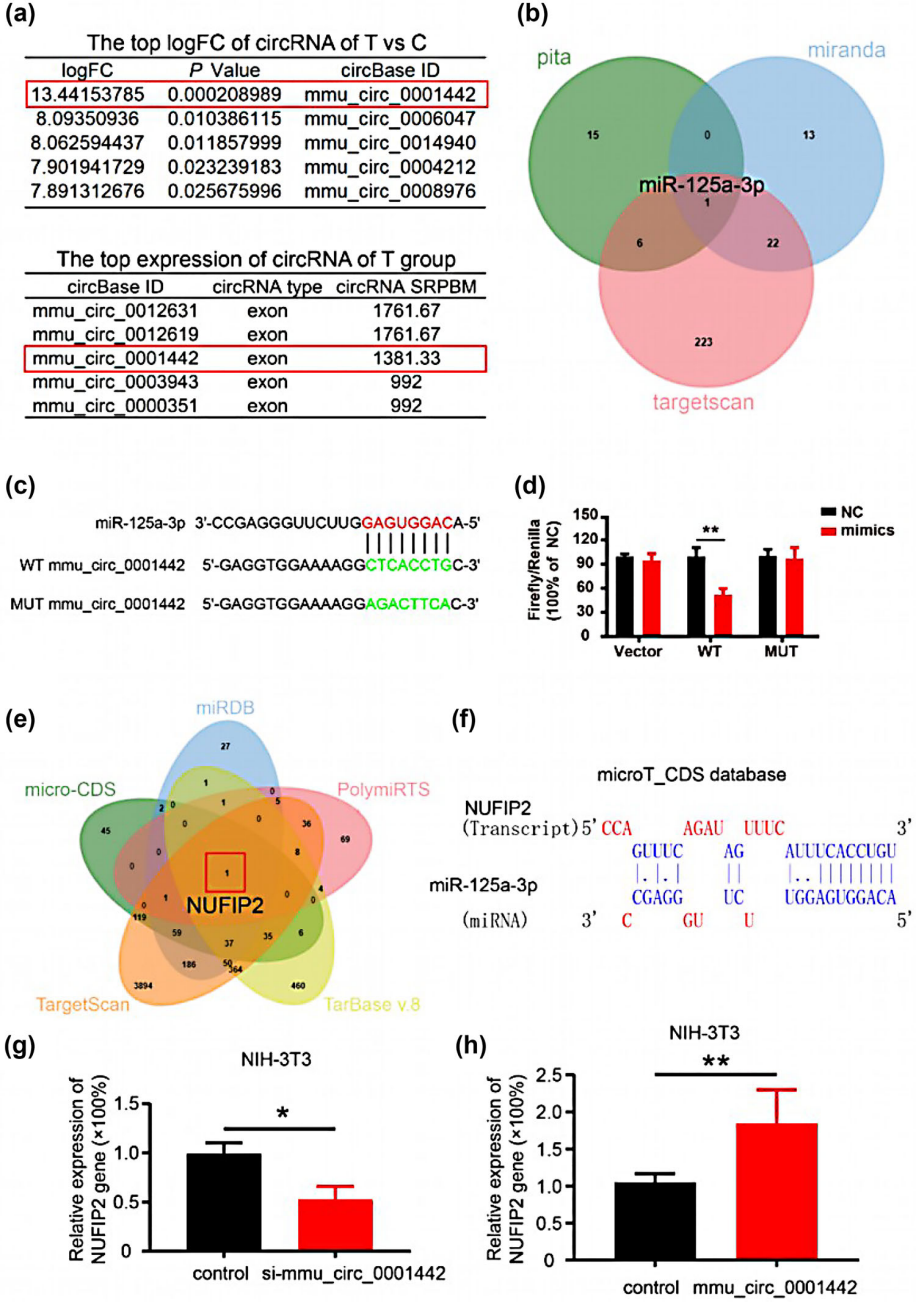
mmu_circ_0001442 was the primary circular RNA (circRNA) responsive for dihydrotestosterone (DHT) stimulation. (a) the target circRNA was confirmed by analyzing the top log FC of circRNA and the top expression of circRNA. (b) miR‐125a‐3p was the downstream responsive for mmu_circ_0001442 after analyzing pita, miRanda, and TargetScan database. (c) the wild type and mutant of mmu_circ_0001442 were designed. (d) the fluorescence intensities of firefly and *Renilla* were calibrated by NC (*N* = 3). (e and f) Target gene prediction analysis indicated that NUFIP2 was the primary response gene for the mmu_circ_0001442/miR‐125a‐3p axis. (g and h) double luciferase reporter gene assay detected the activity of NUFIP2 gene in NIH‐3T3 cells. All values are described as the mean ± SD. *p < .05, **p < .01.

Then, circRNA sequencing was performed to further explore the mechanism and effect of DHT on the hippocampus tissue of representative mice. The differentially expressed circRNA between the T group (DHT pretreatment group) and the C group was screened using SRPBM method. A total of 63 circRNA with significantly difference were confirmed by the standard including expression difference fold greater than 2 and false discovery rate less than 0.05 (Figure 2a). These differentially expressed circRNA consisted of 45 upregulated circRNA and 18 downregulated circRNA (Figure 2a). In addition, the differentially expressions between the T group (DHT pretreatment group) and the C group were compared using heatmap (Figure 2a). As shown in Figure 2b,c, GO and KEGG enrichment analyses were used to look for target genes regulated directly or indirectly by differentially expressed circRNA. Finally, it was confirmed that mmu_circ_0001442 was the primary circRNA responsive for DHT stimulation in PND mice (Figure 3a).

### 3.3 The activation of mmu_circ_0001442/miR‐125a‐3p/NUFIP2 axis

And then some databases, including miRanda, pita, and TargetScan, were preformed to predict the regulatory relationship of mmu_circ_0001442. The downstream regulatory miR‐125a‐3p of mmu_circ_0001442 was confirmed using Venn analysis (Figure 3b). In order to validate the interaction between mmu_circ_0001442 and miR‐125a‐3p, the dual‐luciferase reporter vector containing the WT or MT of mmu_circ_0001442‐3′ UTR sequence and the mimics of miR‐125a‐3p were both constructed for the double luciferase reporter gene assay (Figure 3c). As shown in Figure 3d, the dual‐luciferase reporter gene assay suggested that compared with the NC group, the luciferase activity of the miR‐125‐3p mimics and WT‐mmu_circ_0001442‐3′ UTR co‐transfection group was significantly decreased, whereas miR‐125‐3p mimics did not affect the luciferase activity of MT‐mmu_circ_0001442‐3′ UTR. The convincing result suggested that miR‐125‐3p directly bound to mmu_circ_0001442‐3′ UTR. As shown in Figure 3e,f, the result of target gene prediction analysis indicated that NUFIP2 was the primary response gene for the mmu_circ_0001442/miR‐125a‐3p axis. qPCR assay further clarified the regulatory effect of mmu_circ_0001442 on NUFIP2 gene in NIH‐3T3 cells (Figure 3g,h).

### 3.4 DHT inhibited the LPS‐induced neuroinflammation via activating mmu_circ_0001442/miR‐125a‐3p/NUFIP2 axis in vitro

**FIGURE 4:**
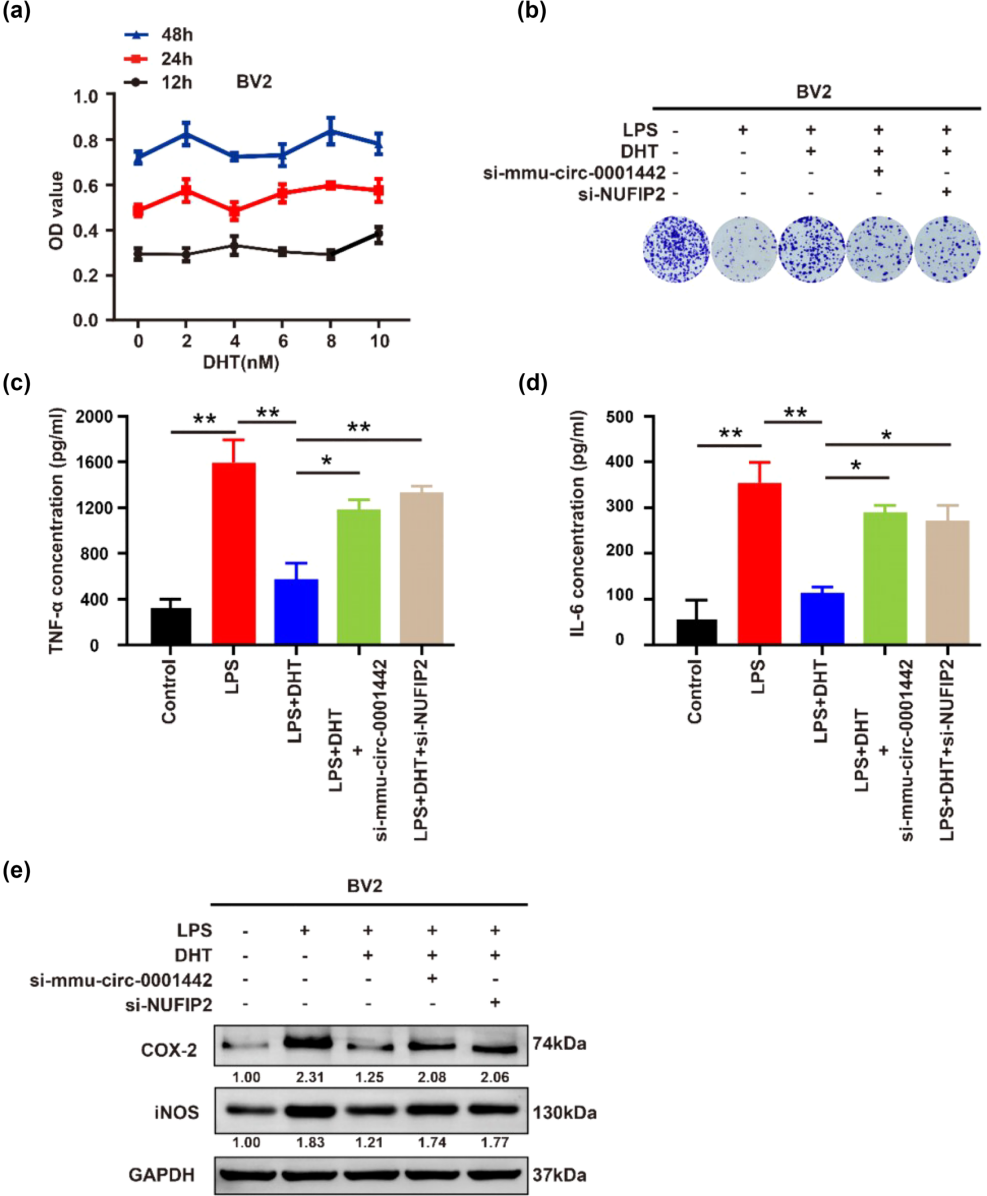
Dihydrotestosterone (DHT) abrogated the lipopolysaccharide (LPS)‐induced inflammation in BV2 cells. (a) after treating BV2 cells with different concentration of DHT for different intervention times, the cell activity of BV2 cells was detected. (b) The plate clone formation assay to measure BV2 cells proliferation. (c and d) ELISA kit: the concentrations of proinflammatory cytokines including IL‐6 and TNF‐α in the BV2 cells culture supernatant were detected after the treatment of DHT or LPS. (e) Western blotting: the expressions of cyclooxygenase‐2 (COX‐2) and inducible nitric oxide synthase (iNOS) protein were detected. All values are described as the mean ± SD. *p < .05, **p < .01.

REDOX damage can promote the expression of multiple proinflammatory cytokines resulting in neurodegenerative diseases (Hsieh & Yang, 2013). We knew that DHT can inhibit REDOX damage and neuroinflammation in PND mice based on above results. To further clarify the effect of DHT on anti‐inflammation in the nervous system, LPS was used to induce the inflammatory response in BV2 cells. The CCK‐8 assay suggested that DHT had no significant effect on the cytoactive of BV2 cells but could inhibit the toxic effect of LPS on BV2 cells (Figure 4a,b). Notably, mmu‐circ‐0001442 and NUFIP2 expressions specifically suppressed by RNA interference can be attenuated by the effect of DHT (Figure 4a,b). The concentrations of proinflammatory cytokines including IL‐6 and TNF‐α in the cell culture supernatant were detected using ELISA kit. The results showed that the concentration of IL‐6 and TNF‐α in the culture supernatant of BV2 cells in the LPS group was significantly increased; but DHT stimulation significantly attenuated the effect of LPS on promoting the expression of IL‐6 and TNF‐α (Figure 4c,d). In addition, RNA interference of mmu‐circ‐0001442 and NUFIP2 expression also can be attenuated the effect of DHT (Figure 4c,d). Likewise, Western blotting results suggested that RNA interference of mmu‐circ‐0001442 and NUFIP2 expression can attenuated the protective effect of DHT in REDOX damage caused by LPS stimulation, manifested as the increased expression of COX‐2 and iNOS (Figure 4e). In a word, DHT stimulation significantly inhibited the promotion effects of LPS on neuroinflammation via activating mmu_circ_0001442/miR‐125a‐3p/NUFIP2 axis in BV2 cells.

## 4 DISCUSSION

At present, the specific pathophysiological mechanism of PND is not fully understood, but increasing evidences suggest that neuroinflammation plays a key role in the development of it (Liu et al., 2022). On the one hand, aberrantly activated microglia is neurotoxic through activating inflammatory signaling nuclear factor‐kappa B or mitogen‐activated protein kinase (MAPK) (Liu et al., 2020). The production of proinflammatory cytokines, such as TNF‐α, IL‐6, and IL‐1β, further aggravates the neuroinflammatory response and leads to cognitive disorder (Plastira et al., 2020). On the other hand, aberrantly activated microglia can promote the production of free radicals such as reactive oxygen species and reactive nitrogen species (Park et al., 2015). These active substances play an important role in the immune response which can adversely affect the function of brain neurons. Therefore, inhibiting the activity of microglia to reduce neuroinflammation and REDOX damage is beneficial to protect postoperative neurocognitive function.

The process of surgery can induce a systemic inflammatory response by promoting the release of local and systemic inflammatory cytokines and the activation of inflammatory signaling molecules (Lin et al., 2000). Local tissue damage caused by surgical operations can promote the release of proinflammatory cytokines such as TNF‐α and IL‐6 from vascular endothelial cell and macrophage at the injury site, thereby causing a cascade effect of inflammatory signals (Hu et al., 2018). It has been reported that IL‐6 and TNF‐α in peripheral serum of patients with PND were significantly increased (Hu et al., 2018; Mathew et al., 2007). Therefore, these studies suggest that surgical trauma is positively associated with the aberrant activation of immune cells especially in microglia (Feng et al., 2017). Subsequently, and the high expression of proinflammatory cytokines on account of the aberrantly activated microglia is positively associated with the severity of neurocognitive impairment.

Androgens mainly include testosterone and its metabolite DHT. According to whether ARs are combined, the effect of androgens can be divided into two models including AR‐dependent activation and AR‐independent activation. AR‐dependent activation means that the binding of androgens to AR to trigger the transcription of target genes, thereby regulating cell viability and differentiation; and AR‐independent activation means that androgens directly activate PI3K, MAPK, and other related signaling pathways to regulate cell growth and metabolic functions. Neuroinflammation is the key etiology of neurodegenerative diseases. Notably, DHT has anti‐neuroinflammatory and neuroprotective effects (Yang et al., 2020). However, the effect of DHT on reducing the occurrence of PND is controversial. Current research suggested that decreased DHT production increases risk of neurodegenerative diseases in elderly men (Drummond et al., 2009; Sumien et al., 2021). On the one hand, there are some clinical observations found that DHT supplementation has positive effect in improving cognitive function (Cai & Li, 2020; Kluger et al., 2020). On the other hand, some clinical studies reported that DHT has anti‐inflammatory properties in peripheral inflammation‐related disease (Mohamad et al., 2019). However, it remains unclear whether DHT has anti‐inflammatory and protection effects in patients with PND. Therefore, in‐depth study of the protective effect and mechanism of DHT on PND has important clinical application value for preventing the occurrence of PND in elder.

## 5 CONCLUSIONS

In summary, this article demonstrated that DHT can inhibit REDOX damage and neuroinflammation by activating the mmu_circ_0001442/miR‐125a‐3p/NUFIP2 axis, thereby alleviating the occurrence of cognitive impairment in mice with perioperative neurocognitive impairment.

## AUTHOR CONTRIBUTIONS

Huaigen Zhang conceived and designed the experiment. Mei Liu, Daying Zhang, and Zhiping Song conducted experiments and wrote the manuscript. Li Liu participated in discussions and proofreading the manuscript. All authors have reviewed and approved the final manuscript version.

## CONFLICT OF INTEREST STATEMENT

All authors declare that they have no conflicts of interests.

## FUNDING INFORMATION

This article was not supported by any funding.

### PEER REVIEW

The peer review history for this article is available at https://publons.com/publon/10.1002/brb3.3180.

## Data Availability

To review raw data of GSE216016 in GEO database: Go to https://www.ncbi.nlm.nih.gov/geo/query/acc.cgi?acc=*GSE216016*, and enter token *ctsvmuaqhloxdwv* into the box.
